# Omics-Driven Biotechnology for Industrial Applications

**DOI:** 10.3389/fbioe.2021.613307

**Published:** 2021-02-23

**Authors:** Bashar Amer, Edward E. K. Baidoo

**Affiliations:** ^1^Lawrence Berkeley National Laboratory, Joint BioEnergy Institute, Emeryville, CA, United States; ^2^Biological Systems and Engineering, Lawrence Berkeley National Laboratory, Berkeley, CA, United States; ^3^U.S. Department of Energy, Agile BioFoundry, Emeryville, CA, United States

**Keywords:** systems biology, genomics, transcriptomics, metabolomics, proteomics, multi-omics, metabolic engineering, biotechnology

## Abstract

Biomanufacturing is a key component of biotechnology that uses biological systems to produce bioproducts of commercial relevance, which are of great interest to the energy, material, pharmaceutical, food, and agriculture industries. Biotechnology-based approaches, such as synthetic biology and metabolic engineering are heavily reliant on “omics” driven systems biology to characterize and understand metabolic networks. Knowledge gained from systems biology experiments aid the development of synthetic biology tools and the advancement of metabolic engineering studies toward establishing robust industrial biomanufacturing platforms. In this review, we discuss recent advances in “omics” technologies, compare the pros and cons of the different “omics” technologies, and discuss the necessary requirements for carrying out multi-omics experiments. We highlight the influence of “omics” technologies on the production of biofuels and bioproducts by metabolic engineering. Finally, we discuss the application of “omics” technologies to agricultural and food biotechnology, and review the impact of “omics” on current COVID-19 research.

## Introduction

Biotechnology employs biological processes, organisms, or systems to yield products and technologies that are improving human lives (Bhatia, 2018). The use of biological systems to manufacture bioproducts of commercial relevance (i.e., biomanufacturing) is a key component of the biotechnology industry. This manufacturing approach is used by the energy, material, pharmaceutical, food, agriculture, and cosmetic industries (Zhang et al., 2017). The bioproducts made from biomanufacturing processes are typically metabolites and proteins, which can be obtained from cells, tissues, and organs. The biological systems producing these bioproducts can be natural or modified by genetic engineering (Zhang et al., 2017), metabolic engineering (optimizing metabolic networks and pathways for increased production of metabolites and/or by-products), synthetic biology (applying engineering principles to the chemical design of biological systems), and protein engineering (optimizing protein design to develop valuable proteins) (Zhang et al., 2017).

Modern biotechnology-based biomanufacturing started in the early twentieth century with the production of short-chain alcohols and ketones, amino acids, organic acids, and vitamin C by microbial mono-culture fermentation (Zhang et al., 2017). This was followed by the production of antibiotics via microbial fermentation in the 1940s and protein drug production in animal cells and microorganisms in the 1980s (Zhang et al., 2017). With the advent of translational research (e.g., stem cell research) in the 2000s, the bioproducts can now be engineered cells, tissues, and organs (e.g., by stem cell engineering) (Roh et al., 2016; Zhang et al., 2017). In addition to this, advancements in synthetic biology and metabolic and protein engineering have been applied to renewable energy research in the development of advanced biofuel and hydrogen production by engineered microorganisms (Zhang et al., 2017). Research efforts are underway at bioenergy research centers (e.g., US DOE Bioenergy Research Centers) to make biofuels more affordable by coproducing them with renewable bioproducts. This is of great importance, as environmental, geopolitical, and economic factors are reshaping our view of global energy and manufacturing demands (Clomburg et al., 2017). The tools (and ideology) from these approaches have also been leveraged by the food industry to produce artificial food products via synthetic biocatalysts in a sustainable way (Zhang et al., 2017).

Synthetic biology and metabolic engineering can benefit from systems biology approaches, which in turn rely on “omics” technologies to characterize and understand metabolic networks. The considerable amount of knowledge obtained from omics-driven systems biology experiments can be used in the development of synthetic biology tools and the advancement of metabolic engineering. This facilitates the manipulation of complex biological systems toward establishing robust industrial biomanufacturing platforms (Baidoo and Teixeira Benites, 2019).

In this review, we aim to examine the influence of “omics” technologies on biotechnology research. “Omics” techniques were compared to understand their relevance and applicability to biotechnology research, especially in the context of microbial biotechnology, with the aim of facilitating the experimental design of individual “omics” and multi-omics studies. Finally, we compared the trends in “omics” utilization during the last two decades to determine their progression in biotechnology research.

## A Comparison of the Major “Omics” Technologies

**Figure 1:**
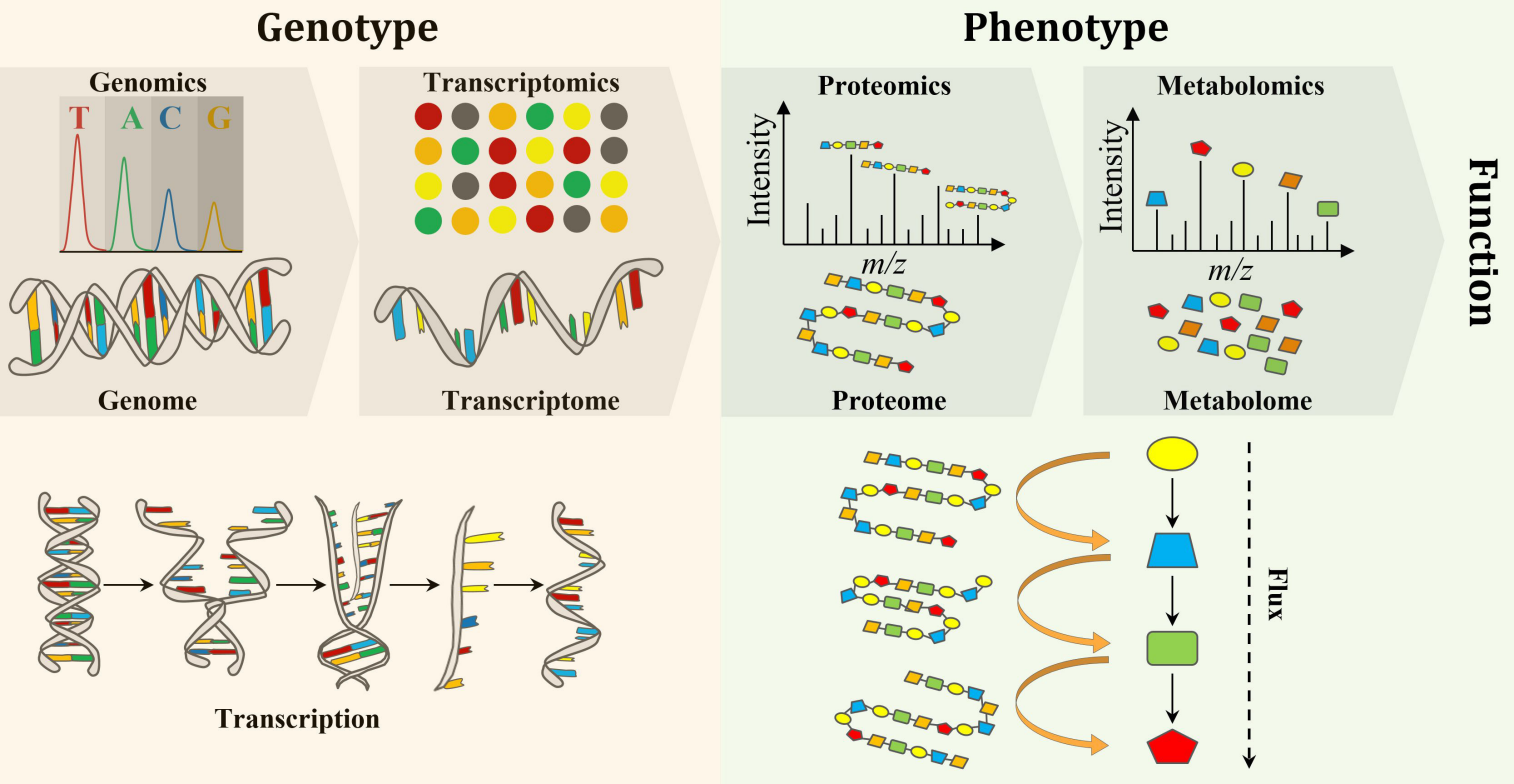
An overview of the flow of molecular information from genes to metabolites to function and phenotype, and the interactions between the “omes” and the “omics” techniques used to measure them.

Biological engineering requires the accurate prediction of phenotype from genotype. Thus, testing and validating modified and synthesized genomes (i.e, genomics) as well as the study of the transcriptome (the complete set of RNA transcripts), which is expressed from the genome (i.e., transcriptomics), are crucial to evaluating genome engineering. Proteomics and metabolomics have also gained a lot of attention due to their provision of metabolic information pertaining to both function and phenotype (Baidoo, 2019). Forty years ago, scientists realized that the flow of biochemical information in biological systems is not unidirectional from the genome to metabolome, but rather a set of interactions between the “omes” (Roberts et al., 2012). Therefore, a multi-omics approach is necessary for the elucidation of chemical structure, function, development, adaptation, and evolution of biological systems for deeper understanding of the principles of life (Baidoo, 2019) (Figure 1).

In comparison to metabolites and proteins, genes are less chemically heterogeneous. Each gene is made up of DNA that is composed of only four basic nucleotides (i.e., guanine, adenine, cytosine and thymine), whereas each protein is composed of a mixture of 32 amino acids, while metabolites are much more variable in their chemical structures (Wang et al., 2010). Therefore, it is analytically less challenging to perform genomics and transcriptomics, when compared to proteomics and metabolomics (Aizat et al., 2018). Consequently, genomics and transcriptomics provide the most comprehensive and robust platforms for biotechnology applications. Over the past few decades, research has shown that genomics and transcriptomics cannot solely provide a complete description of complex biological systems as genetic information can produce more questions than answers. For instance, genomics can describe genes and their interactions (measure genotype) but cannot explain phenotypes. Thus, the attention is turned to the utilization of other “omics” techniques, such as proteomics and metabolomics, which can bridge the gap between genetic potential and final phenotype to facilitate a greater understanding of biological systems (Smith and Figeys, 2006; Wilmes et al., 2015). While transcriptomics (transcription) and proteomics (translation) provide information on gene expression, the latter directly links genotype to phenotype. In addition to providing phenotypic information, the metabolome provides an instant response to genetic and/or environmental perturbations and, therefore, provides a snap shot of the actual metabolic and physiological state of a cell (Tang, 2011). However, metabolomics alone is not able to measure changes at the gene level and correlate them with the observable properties of organisms, the phenotypes, which are produced by the genotype in the first place (Fiehn, 2001). Therefore, a comprehensive understanding of an organism on a molecular level requires the integration of “omics” data in order to discover new molecules and pathways (Wang et al., 2010) (Figure 1). Integration of “omics” data helps to assess the flow of information from one “omics” level to the other and, therefore, links genotype to phenotype (Subramanian et al., 2020). Furthermore, the combination of “omics” techniques is important to address open biological questions (i.e., data driven research) that accelerate our understanding of the system as a whole and boost the use of systems metabolic engineering tools in industrial settings (Zhao et al., 2020).

### Genomics and Transcriptomics

The construction of predictable and preferred phenotypes is crucial in synthetic biology; therefore, tight and tunable control of gene expression is highly desirable. Biological engineering, moreover, is greatly benefiting from the recent innovations in genomics and genome editing technologies, which offer advanced tools to re-engineer naturally evolved systems and to build new systems as well. In addition, advances in *de novo* synthesis and *in vivo* gene targeting enable efficient testing of model-driven hypotheses. Furthermore, genomics allows the high-throughput DNA sequencing and large-scale bimolecular modeling of metabolic and signaling networks in natural and engineered strains (Pagani et al., 2012).

#### Genomics and Transcriptomics Analysis

One of the challenges facing traditional genomics (and other “omics”) analyses is that not all microorganisms can be cultured in a laboratory setting. Furthermore, isolated strains might behave differently in culture than in their natural environments. Therefore, there was an urgent need to develop cultivation independent methods to study microbial communities (VerBerkmoes et al., 2009). Metatranscriptomics can reveal the diversity of active genes within microbial communities (e.g., 16S rRNA sequencing for reconstructing phylogenies) (Bashiardes et al., 2016).

Metagenomics studies the structure and function of genetic material in complex samples of multi-organisms as well as of entire microbial communities without a cultivation step and can offer a solution for such challenges and facilitate the discovery of novel genes, enzymes, and metabolic pathways. Metagenomics analyses are classified as sequence-based and function-based screening, which are used to discover and identify, respectively, novel natural genes and compounds from environmental samples (Chistoserdova, 2010; Gilbert and Heiner, 2015; Kumar Awasthi et al., 2020). For example, metagenomics is actively used in agricultural research to understand the microbial communities in the soil system (Durot et al., 2009), to examine various microbes that can stimulate the cycling of macro- and micro-nutrients, and the release of essential enzymes, which enhance crop production (Cupples, 2005).

Nanopore sequencers are massively parallel sequencing technologies. Oxford Nanopore Technologies (ONT), in particular, provides a single molecule sequencer using a protein nanopore that realizes direct sequencing without DNA synthesis or amplification (Brown and Clarke, 2016; Roumpeka et al., 2017). The ONT sequencer can determine DNA/RNA modifications and sequence an ultra-long read limited by the input nucleotide length (Kono and Arakawa, 2019). However, ONT reads require polishing and great care needs to be taken when contigs are polished individually to avoid the removal of true, natural sequence diversity due to cross mapping of reads in repeat regions. Therefore, it was found that it is crucial to apply long-range information technologies (e.g., 10x genomics, Hi-C, synthetic long reads) and to develop new algorithms to simplify the extensive assembly and polishing workflow (Somerville et al., 2018).

Sort-seq is a single-cell sequencing platform, which combines flow cytometry, binned fluorescence-activated cell sorting (FACS), next-generation sequencing (NGS), and statistical inference to quantify the dynamic range of many biosensor variants in parallel (Rohlhill et al., 2017; Batrakou et al., 2020; Koberstein, 2020). FACS, which enables the sorting of single cells, allows the enrichment of specific cells to generate high-resolution gene expression and transcriptional maps (Kambale et al., 2019). NGS and RNA sequencing (RNA-seq) technologies enable the large-scale DNA and RNA sequencing of the entire genome and transcriptome, respectively, providing an unbiased and comprehensive view of biological systems towards understanding genomic function (Frese et al., 2013; Alfaro et al., 2019; Stark et al., 2019). Examples of NGS platforms include the Illumina HiSeq, Genome Analyzer Systems, 454 Genome Sequencer FLX Titanium System, the Helicos HeliScope, the SOLiD sequencing platform and the Ion Torrent Sequencing platform. In addition, there are other techniques used to measure the interaction between proteins and DNA, such as chromatin immunoprecipitation (ChIP). ChIP followed by NGS sequencing (ChIP-seq) has high potential for detailing the binding sites of various transcription factors and assaying protein–DNA interaction at a whole-genome level (Roukos, 2012).

Bar-seq (barcode analysis by sequencing) is another high-throughput quantitative DNA sequencing technique that enables the parallel phenotyping of pools of thousands of mutants and monitoring thousands of gene-chemical interactions (Smith et al., 2010; Robinson et al., 2014). Techniques, such as bar-seq can lower the complexity of data obtained from a large number of sequence reads, thus, making NGS more efficient and affordable (Smith et al., 2009).

New computational tools have enabled researchers to perform fast and accurate analysis of big genomics data. Extracted genomic information has been used to model metabolic processes and signaling networks across the entire cell, generating many new testable hypotheses (Lewis et al., 2012; Esvelt and Wang, 2013). Due to the robustness of genomic measurements, there are numerous genomics databases and data analysis tools available (Roumpeka et al., 2017).

### Proteomics

Proteomics focuses on the analysis of proteins and peptides produced by cells at different stages of development and life cycle and in biological systems under a given growth condition. Proteomics is also used to elucidate the temporal dynamics of protein expression levels or post-translational modification (PTM) (VerBerkmoes et al., 2009).

#### Proteomics Sample Preparation

High biological sample diversity and complexity and the dynamic range of protein levels present in such samples are the main challenges that proteomics encounter. These factors, in addition to the large number of proteins, complicate the analysis of low abundance proteins. The development of automated sample preparation workflows are becoming more common for high-throughput, quantitative proteomic assays of microbes. One automated workflow was able to quantify >600 peptides with a 15.8% median coefficient of variation, demonstrating the robustness of this approach (Chen et al., 2019). Another high-throughput automatable workflow was developed to increase the yield of lysis of several representative bacterial and eukaryotic microorganisms via vigorous bead-beating with silica and glass beads in presence of detergents (Hayoun et al., 2019). Interestingly, a universal, high-throughput and a detergent-free sample preparation protocol was developed this year for peptide generation from various microbes [i.e., *Escherichia coli* (*E. coli*), *Staphylococcus aureus* and *Bacillus cereus*]. The protocol holds the potential to dramatically simplify and standardize sample preparation while improving the depth of proteome coverage especially for challenging samples (Doellinger et al., 2020).

#### Proteomics Data Acquisition

##### Protein Identification and Structural Elucidation

Most proteomics workflows are based on a bottom-up approach, where protein is extracted, digested (e.g., trypsin digestion) into proteolytic peptides, then analyzed via MS (Kleiner et al., 2017). When liquid-chromatography is coupled to mass spectrometry (LC-MS), both qualitative and quantitative data analysis of proteins are improved. Moreover, the application of multidimensional LC separation prior to MS protein analysis provides enhanced MS sensitivity by reducing sample complexity and increasing the number of chromatographic peaks that can be resolved in a single analytical run (Hinzke et al., 2019; Duong et al., 2020). Targeted proteomics via LC-tandem MS (LC-MS/MS) is a commonly used MS method, where the analysis focuses on a subset of biological proteins of interest (Marx, 2013). When a bottom-up approach is applied to all proteins within a given biological system, it is called shotgun (untargeted) proteomics (Wolters et al., 2001; Nesvizhskii and Aebersold, 2005). Top-down proteomics, conversely, is based on the analysis (via LC-MS or LC-MS/MS) of intact proteins, and thus, provides unique information about the molecular structure of proteins (e.g., PTM) (Catherman et al., 2014). However, it is not always possible to separate intact proteins, especially large proteins, prior to MS analysis in a top-down approach. Besides that, top-down is less sensitive and has a lower throughput than a bottom-up approach (Catherman et al., 2014).

Accurate determination of protein structure helps to define their roles and functions in biological systems. However, many folded proteins have complex structures, which complicates their structural elucidation (Yates, 2019). Therefore, cryogenic electron microscopy and ion-mobility-MS are used to determine the structures of such proteins such proteins (Yates, 2019). Moreover, a combination of MALDI, high resolution MS (i.e., orbitrap and ion trap MS) and a UV–Vis-based reduction assay is used to elucidate peptide modification via the analysis of specific fragmentation of synthesized peptides, which might have inhibitory effects on various diseases (Rühl et al., 2019).

The identification of PTM peptides can be difficult in the case of labile modifications (e.g., phosphorylation and S-nitrosylation) that might break down during MS/MS fragmentation. Such modifications require soft fragmentation and high-resolution methods to identify and determine the location of a PTM. Electron-transfer dissociation is considered to be the favorable choice for the identification of liable PTM as it transfers electrons to multi-protonated proteins or peptides, which leads to N-Cα backbone bond cleavage (Chen et al., 2017).

Metaproteomics is the large-scale characterization of the entire protein complement of environmental microbiota at a given point in time to determine structure (Wilmes and Bond, 2004; Kleiner et al., 2017), metabolism and physiology of community components (Kleiner et al., 2012). The recent advancement in LC and high-resolution MS have enabled the identification and quantification of more than 10,000 peptides and proteins per sample in metaproteomics (Kleiner, 2019). Metaproteomics can also measure interactions between community components (Hamann et al., 2016) and assess substrate consumption (Bryson et al., 2016; Kleiner et al., 2018).

##### Protein Quantification

Besides identification, MS-based technologies became the tools of choice for the quantification of proteins in an organism (Karpievitch et al., 2010). Stable isotope labeling is one approach that can be used to quantify proteins by measuring the relative abundance of labeled protein to non-labeled protein (VerBerkmoes et al., 2009). However, the variation in ionization efficiency among peptides and proteins and the low recovery of some peptides (e.g., hydrophobic peptides adhere to surfaces) can affect the accuracy of their direct quantification. Recent advances in MS acquisition rate, detection, and resolution have addressed much of the sensitivity concerns of MS-based quantification for proteomics (Iwamoto and Shimada, 2018). MS sensitivity was further enhanced with the application of micro-flow (Krisp et al., 2015; Bian et al., 2020) and nano-flow (Wilson et al., 2015) LC-MS. Another major advancement for global protein quantification was the introduction of isobaric tags or multiplexed proteomics, which in a single experiment enables the quantification of proteins across multiple samples (Pappireddi et al., 2019). Tandem-mass-tags are examples of commonly used isobaric tags for instance in human cerebrospinal fluids (Dayon et al., 2008).

#### Proteomics Data Analysis

Proteomics data analysis tools are generally used for protein identification (via bioinformatics) and quantification, and bioinformatics techniques tools used to process the proteomics data. A few examples of data analysis tools that are used for the identification of peptides and proteins include Mascot (Eng et al., 1994), Swiss-Prot (Bairoch and Boeckmann, 1994), Sequest (Perkins et al., 1999), Tandem (Craig and Beavis, 2004), Skyline (MacLean et al., 2010), Uni-Prot,^1^ UniNovo (Jeong et al., 2013), and SWPepNovo (Li et al., 2019). Such algorithm-based software were developed to match the MS collected data from peptide/protein analysis to their base peptides/proteins and with *in silico* predicted intact masses and fragmentation patterns (Urgen Cox and Mann, 2011). Moreover, they determine both the mass and exact location of any possible modifications (Hansen et al., 2001; Savitski et al., 2006). Common bioinformatics techniques tools for proteomics data analysis include CRONOS (Waegele et al., 2009), COVAIN (Sun and Weckwerth, 2012), SIGNOR (Perfetto et al., 2016), KEGG (Kanehisa et al., 2017), and STRING v11 (Szklarczyk et al., 2019).

### Metabolomics

Metabolomics, which is the measurement of small molecule substrates, intermediates, and/or end products of cellular metabolism (i.e. metabolites), provides an immediate and dynamic response to genetic and/or environmental perturbations in a biological system (Fiehn, 2002; Ellis and Goodacre, 2012; Zhao et al., 2020). Targeted and untargeted metabolomics are used to quantify a group of defined metabolites and determine all measurable metabolites in a biological sample, respectively (Scalbert et al., 2009). MS-based metabolomics, like proteomics, normally employs separation [e.g. LC and gas chromatography (GC)] or capillary electrophoresis (CE) before MS detection (Fiehn, 2002). Whereas, MALDI-MS conducts high-throughput screening without separation.

Nuclear magnetic resonance (NMR) spectroscopy is a powerful analytical technique for high-throughput metabolic fingerprinting and provides more reliable metabolite structure (e.g., via 2D NMR) identification than MS (Giraudeau, 2020). However, although NMR offers unambiguous structure determination of unknown metabolites via ^1^H- and ^13^C-NMR, MS-based methods comprise widely accessed metabolomics techniques due to higher sensitivity and lower instrumentation cost (Chatham and Blackband, 2001). Furthermore, NMR is semi-quantitative whereas MS is quantitative, thus, NMR and MS are highly complementary techniques. In addition, the diverse physiochemical properties (e.g., solubility, reactivity, stability, and polarity) of the metabolome limits our ability to analyze all metabolites from a biological system with a single or even a limited-set of analytical techniques (Fiehn, 2002). Therefore, multiple methods are used for comprehensive metabolome characterization.

#### Metabolomics Sample Preparation

Metabolites are constantly going under reformation and transformation in biochemical reactions within a cell and/or being thermally degraded (and in some cases oxidized) at ambient conditions (Scalbert et al., 2009). Therefore, quick and efficient metabolic quenching protocols are required to accurately quantify metabolic information. Not surprisingly, researchers tend to develop metabolic quenching methods in conjunction to and metabolite extraction protocols. Doran et al. (2017), for example, proposed an acidic-based metabolic quenching to aqueous-alcohol metabolite extraction. This protocol yielded low metabolite leakage and high extraction recovery in *Acidithiobacillus ferrooxidans*. Complex biological sample matrices can also suppress metabolite MS detection. Thus, clean up strategies, such as solid phase extraction (SPE) and solid phase micro-extraction (SPME) can reduce the complexity of sample matrices prior to LC-MS and GC-MS analysis, thereby increasing the quantitative capability of metabolomics methods (Yang et al., 2011). The last 5 years witnessed the development of high-throughput 96-well plate SPE (Li et al., 2015) and 96-well automated SPME (Mousavi et al., 2015) for the simultaneous extraction of metabolites and lipids from biological samples.

In addition, robotics and microfluidics tools can be applied to high-throughput synthetic biology applications by automating cell preparation and metabolite extraction to increase coverage (Yizhak et al., 2010; Koh et al., 2018; Vavricka et al., 2020). Automated liquid handler technologies, therefore, are important for high-throughput sample preparation as they ensure good quality and reproducibility of sample extraction and processing for unbiased measurement of metabolic differences (e.g., based on disease states or interventions stimuli) (Liu et al., 2019).

#### Metabolomics Data Acquisition

The development of nanoelectrospray-ionization and direct infusion nanoelectrospray high-resolution MS have led to a considerable increase in the dynamic range and detection sensitivity of metabolites from tissues and biofluids in human studies (Chekmeneva et al., 2017; Southam et al., 2017). Generally, nanospray technology is more sensitive than electrospray, but suffer from low robustness. However, nanoelectrospray employs low level of nebulization and flow rate to achieve high sensitivity without compromising robustness (Guo et al., 2016). Besides, the application of ion mobility and high resolution MS has improved the identification of isomers, thereby enabling a more accurate assessment of their biological roles (Ren et al., 2018; Rathahao-Paris et al., 2019). Moreover, new developments in orbitrap MS systems have improved metabolites annotation and coverage in GC- and LC-MS studies (Simirgiotis et al., 2017; Misra et al., 2018; Manier et al., 2019; Stettin et al., 2020).

Although GC-MS requires more sample preparation steps when derivatizing hydrophilic non-volatile metabolites, it is more robust than LC-MS. Moreover, method development is easier for GC-MS than for LC-MS. GC-MS also achieves better identification of untargeted metabolites due to standardized ionization conditions, which makes it possible to set up a universal compound identification library/database, such as NIST. While CE achieves the highest separation efficiency, CE-MS is the least robust and sensitive of the three separation techniques.

Real-time metabolomics enables the simultaneous and high-throughput analysis of microbial metabolites without the need for time-consuming sample preparation steps (Link et al., 2015; Boguszewicz et al., 2019; Nguyen et al., 2020). However, the lack of chromatographic or electrophoretic separation in this approach reduces the quantitative capability of this technique (Baidoo and Teixeira Benites, 2019). While MALDI can be used for high-throughput metabolite screening, MALDI imaging MS has emerged as a powerful tool for analyzing tissue specimen in an unprecedented detail. MALDI imaging MS has made significant contributions to the understanding of the biology of disease and its perspectives for pathology research and practice, as well as in pharmaceutical studies (Aichler and Walch, 2015; Mahajan and Ontaneda, 2017; Schulz et al., 2019).

Metabolomics technologies are regularly applied to metabolic flux analysis (MFA, i.e., ^13^C) studies (Baidoo and Teixeira Benites, 2019). MFA determines the rates of *in vivo* metabolic reactions. Thus, enabling an understanding of carbon and energy flow throughout the metabolic network in a cell. Overall, MFA accelerates the discovery of novel metabolic pathways and enzymes for improved synthetic bioproduction (Feng et al., 2010; Ando and García Martín, 2019; Babele and Young, 2019; Vavricka et al., 2020). However, the availability and high cost of stable isotope compounds can limit MFA capability (Gonzalez and Pierron, 2015).

#### Metabolomics Data Analysis

Multivariate data analysis methods, such as principal component analysis (PCA) and partial least squares (PLS) analysis are used to analyze large quantities of metabolic profiling data (i.e., reveal clustering-based on features). In addition, there is a need for advanced pathway analysis tools to interpret metabolomics data to solve some of the most challenging biological paradoxes and reveal optimal conditions for biological systems. Such techniques enable systems biology researchers to utilize metabolomics data as a resource that contributes to an iterative cycle of hypothesis generating and hypothesis testing phases (Kell, 2004; Vavricka et al., 2020). To address all of this, more attention is being paid to the area of big data and machine learning. Thus, the state-of-the-art understanding of cell metabolism can be improved and further combined with mechanistic models to automate synthetic biology and intelligent biomanufacturing (Oyetunde et al., 2018). To this end, recent advancements in metabolomics tools for data analysis, storing and sharing have been developed [e.g., WebSpecmine (Cardoso et al., 2019), SIRIUS 4 (Dührkop et al., 2019), MetaboAnalyst 4.0 (Chong et al., 2018), and SECIM (Kirpich et al., 2018)]. Knowledge of biology (e.g., regulation, metabolism, physiology, etc.) is still, however, necessary for efficient experimental design and accurate data interpretation in order to understand and accurately characterize biological systems.

### Multi-Omics for Systems Biology

The recent advancement in omics technologies has improved the analysis efficiency by reducing cost and time, but also by collecting informative and meaningful multi-omics data. Thus, facilitating the implementation of multi-omics techniques in systems biology studies. However, integrating multi-omics platforms is still an ongoing challenge due to their inherent data differences (Saito and Matsuda, 2010; Yizhak et al., 2010; Brunk et al., 2016; Koh et al., 2018; Pinu et al., 2019; Vavricka et al., 2020). For example, genomics data are qualitative, accurate and reproducible, while other “omics” data, such as proteomics and metabolomics are both qualitative and quantitative, not as reproducible, and noisy (Kuo et al., 2002; MacLean et al., 2010; Guo et al., 2013; Gross et al., 2018). Further, multi-omics data is normally pre-treated by various data treatment methods (e.g., deconvolution, normalization, scaling, and transformation) and software before being integrated. Multi-omics studies also require experts in their respective “omics” fields (as well as IT support) to validate multi-omics data. While this provides greater data interpretation accuracy it does, however, complicate data acquisition and analysis.

Recently, Pinu et al. discussed some recommendations to overcome the major challenge facing the implementation of multi-omics techniques in systems biology, which is the differences among their inherent data. The aim of their recommendations is to make researchers aware of the importance of having a proper experimental design in the first place. Thus, the appropriate biological samples should be carefully selected, prepared, and stored before planning any “omics” study. Afterward, researchers should carefully collect quantitative multi-omics data and associated meta-data and select better tools for integration and interpretation of the data. Finally, develop new resources for the deposition of intact multi-omics data sets (Pinu et al., 2019). It is also necessary to select or develop methods that keep the optimum balance between high recovery and low degradation of extracted biological features.

As scientists are becoming more aware of the importance of multi-omics analysis, a number of tools, databases, and methods are being developed for the aim of integrating multi-omics data sets. These tools perform advanced statistics (e.g., multivariate data analysis) and data illustration (e.g., correlation maps). Examples of databases used for multi-omics analysis include ECMDB 2.0 (Sajed et al., 2016), *Saccharomyces* Genome Database (MacPherson et al., 2017), YMDB 2.0 (Ramirez-Gaona et al., 2017), GenBank (Benson et al., 2013), KEGG (Kanehisa and Subramaniam, 2002), and many others. A recent review by Subramaniam et al. showed that common multi-omics data integration and interpretation tools were able to derive new insights from data, conduct disease subtyping, and obtain diagnostic biomarker prediction (Subramanian et al., 2020).

Table 1 provides a comprehensive comparison of the major “omics” technologies. The aim of this comparison is to facilitate the experimental design of individual “omics” and multi-omics studies by highlighting the general characteristics of each technology.

**Table 1:** A comparison of the major “omics” techniques.

**Technique**	**Genomics**	**Transcriptomics**	**Proteomics**	**Metabolomics**	**Multi-omics**
Study	- Genome (the complete set of genes in a biological system) - Genome sequence - Gene functions and interactions - Metagenomics	- Transcriptome (the complete set of RNA transcripts in a biological system) - Gene transcription (i.e., gene expression) - Transcriptome sequence - Metatranscriptomics via 16S rRNA	- Proteome (the complete set of proteins in a biological system) - Protein translation (i.e., gene expression) - Post-translational modification (PTM) state of proteins - Metaproteomics	- Metabolome (the complete set of metabolites in a biological system) - Metabolites (i.e., substrates, intermediates or end products of cellular metabolism) - Pathway flux (i.e., concentration and/or metabolic flux analysis)	- Integrated information of genes, transcriptomes, proteins, and metabolites
Advantages	- Evaluate genome modification in engineered vs. naturally evolved systems	- Evaluate gene function via mRNA transcripts - 16S rRNA sequencing for reconstructing phylogenies	- Assess gene function - Evaluate protein translation - Evaluate PTM - Identify diagnostic biomarkers - Provides phenotypic information	- Assess gene function - Identify metabolic pathway bottlenecks - Identify diagnostic biomarkers (e.g., productivity biomarkers) - Evaluate protein function - Provides phenotypic information	- Identify diagnostic biomarkers with a high degree of accuracy - Provides a comprehensive knowledge and understanding of biological systems
Challenges/ Disadvantages	- Cannot solely provide complete description of complex biological systems (i.e., cannot describe phenotypes)	- Cannot solely provide complete description of complex biological systems (i.e., cannot describe phenotypes) - Insufficient information due to PTM - Cross contamination and cross hybridization	- High instrument cost - Difficult protein/peptide quantification - Inaccurate analysis of labile PTM - Can be expensive as it requires advanced tools (e.g., mass spectrometry) - Low abundance proteins are difficult to analyze - Cross contamination during enzymatic proteolysis (same peptide may come from different proteins) - Difficult to cover whole proteome due to large number of proteins	- High instrument cost - The metabolome is very chemically diverse - Metabolites can have short half-lives due to instability and/or bio-transformations - Low abundance metabolites are difficult to analyze - Challenging sample preparation (e.g., metabolite extraction, and matrix clean up) - Difficult to identify source of metabolite production and consumption in microbial communities	- High data volume and complexity - This approach can be very expensive - Requires good/rigorous experimental design that accounting for all parameters pertaining to individual and combined “omics” technologies - Requires advanced data integration and analysis tools, and specialists from each discipline
Relative throughput	- Highest (fast DNA sequencing)	- High (fast RNA sequencing)	- Moderate	- Moderate	- Depends on selected “omics”
Ideal for	- Testing model-driven hypotheses (targeted approach)	- Testing model-driven hypotheses (targeted approach)	- Targeted (i.e., bottom-up) - Untargeted (i.e., shotgun) - Identifying pathway bottlenecks	- Targeted analysis - Untargeted analysis - Identifying pathway bottlenecks	- Understanding biological systems - Identify diagnostic biomarkers - Identify bioproduction bottleneck
Pathway analysis	- No	- No	- Yes (e.g., via protein abundance/quantity and PTM)	- Yes (e.g., via the assessment of protein function and metabolite production)	- Yes (integrated proteomics and metabolomics)
Relative coverage	- Very comprehensive	- Comprehensive	- Good	- Moderate	- Depend on selected “omics” techniques
Information gained	- Genotype	- Genotype	- Phenotype	- Phenotype	- Connects genotype to phenotype
Type of data	- Qualitative	- Qualitative and quantitative	- Qualitative and quantitative	- Qualitative and quantitative	- Qualitative and quantitative
Relative ease of sample preparation	- Easiest (chemically homogeneous, and stable, thus, easy sample preparation, storage and analysis)	- Easy to moderate	- Moderate (proteins are chemically heterogeneous, and moderately stable, thus, challenging sample preparation, and moderately hard to store)	- Difficult (metabolites are physio-chemically heterogeneous, and unstable (e.g., thermally labile), thus, challenging sample preparation (e.g., metabolite extraction, and matrix clean up), and storage)	- Most difficult
Relative ease of data acquisition	- Easiest	- Easy	- Moderate to difficult	- Difficult (no single analytical technique, nor multiple analytical techniques (e.g., GC-MS, LC-MS, CE-MS, and NMR) can cover the whole metabolome)	- Depends on selected “omics” techniques
Key acquisition tools	- Next generation sequencing (NGS) - PCR - RFLP-PCR	- RNA sequencing (RNA-seq)	- LC-MS (e.g., orbitrap, and TOF) - MALDI	- LC-MS - GC-MS - CE-MS - NMR	- Combination of “omics” acquisition tools
Relative ease of data analysis	- Easiest (fast and accurate data analysis)	- Easy	- Difficult (protein identification and quantification are challenging steps)	- Difficult (data need pre-treatment (e.g., normalization, scaling, and transformation) before analysis)	- Most difficult (integration of various “omics” data is challenging)
Key data analysis tools	- EBI - GEO - ArrayExpress - GenBank	- DESeq2 - DEXseq	- Mascot, Sequest - Tandem - Skyline - Uni-prot, Swiss-prot	- R and Matlab based tools - SIMCA - WebSpecimine, SIRIUS 4 - MetaboAnalyst 4.0, SECIM - COLMAR, MzMine	- ECMDB 2.0 - YMDB 2.0 - GenBank
Overall relative ease of analysis	- Easiest	- Easy	- Moderate to difficult	- Difficult	- Most difficult
Reproducibility	- High	- Good	- Moderate	- Low to moderate	- Depend on selected “omics” techniques

## Omics-Guided Biotechnology

**Figure 2:**
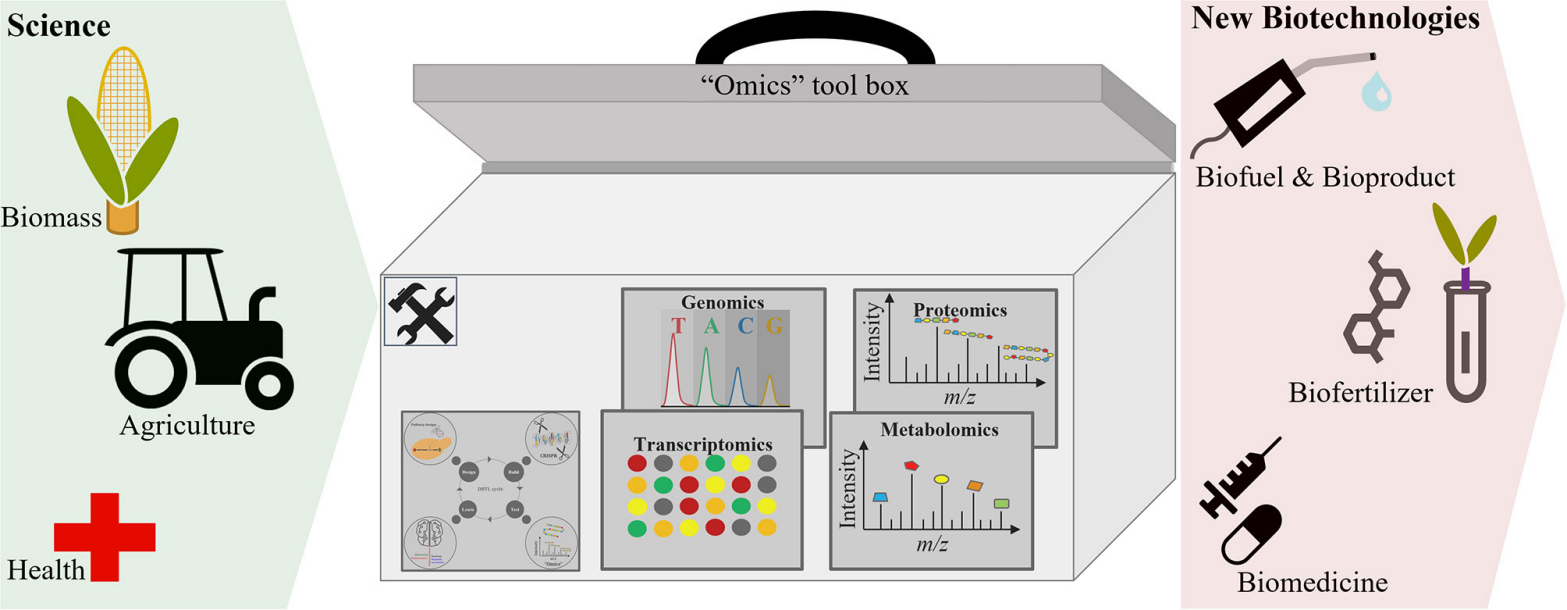
“Omics” approaches facilitate the development of new biotechnologies.

“Omics” technologies are becoming increasingly involved in the development of biotechnological processes for the production of many substantial products. The use of “omics” technologies to characterize and understand biological systems has enabled researchers to select and predict phenotypes (Abid et al., 2018; Babar et al., 2018), which aids the optimization of biotechnological processes toward enhanced production (in quality and quantity) of commercially relevant products (Figure 2). This section discusses the application of “omics” in the development of biofuels and bioproducts, agricultural biotechnology, food biotechnology, and bio-therapeutics. In addition, this section discusses the involvement of “omics” technologies in the development of bio-therapeutics for COVID-19.

### Omics-Guided Metabolic Engineering of *E. coli* and Yeast Toward the Production of Primary and Secondary Metabolism-Based Biofuels and Bioproducts

Microbial production of bio-based chemicals represents an appealing and more sustainable alternative to traditional petrochemicals (Opgenorth et al., 2019) and has led to a growing catalog of natural products and high-value chemicals (Carbonell et al., 2018). The use of lignocellulosic biomass offers an economical approach to generate biofuels and bioproducts (Fatima et al., 2018). However, to achieve consistent conversion of low-cost input material into value-added products (Yan and Fong, 2018) at industrial levels requires systematic engineering workflows.

**Figure 3:**
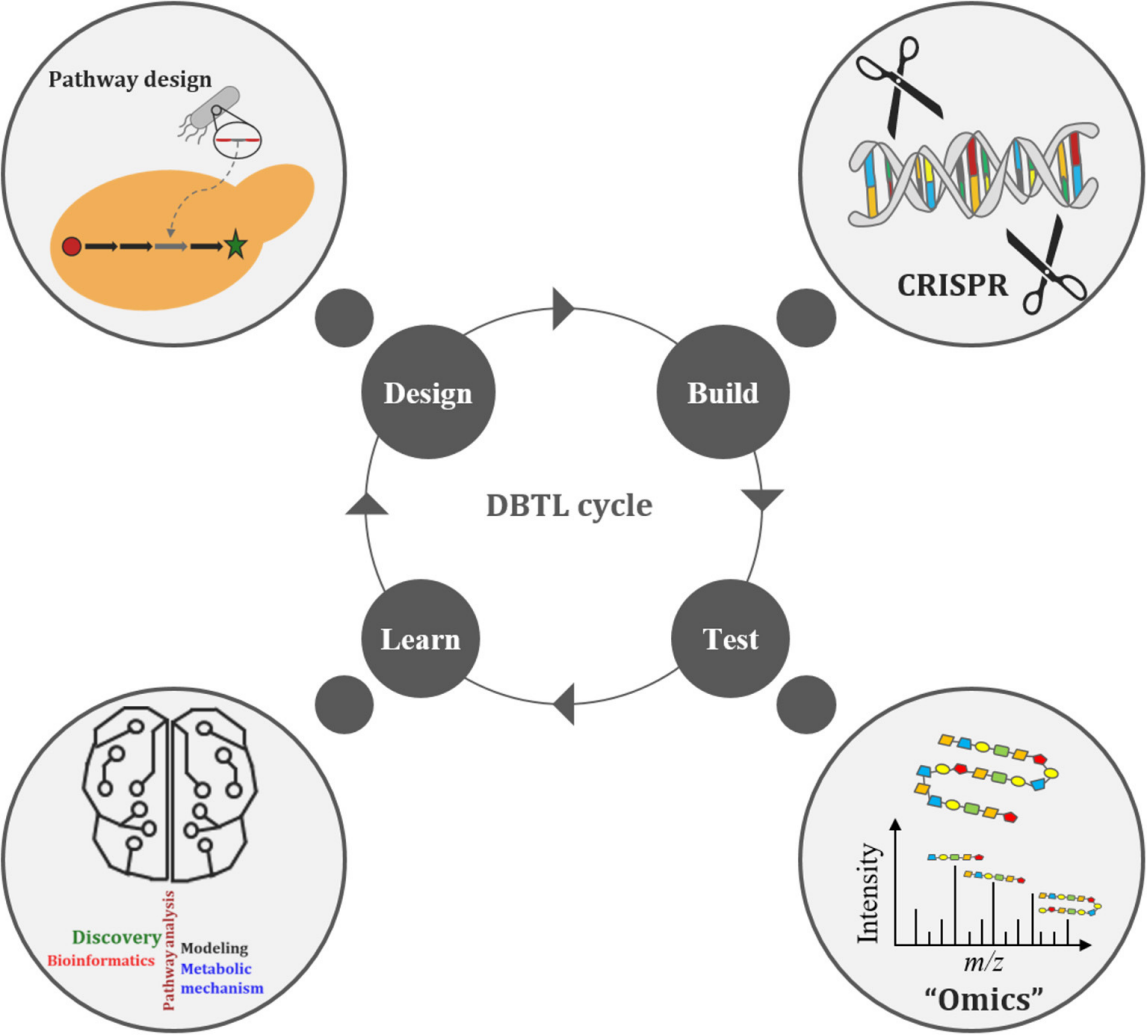
The Design-Build-Test-Learn (DBTL) cycle of metabolic engineering.

The Design-Build-Test-Learn (DBTL) cycle is becoming an increasingly adopted frame-work for metabolic engineering experiments (Opgenorth et al., 2019). It represents a systematic and efficient approach to strain development efforts in biofuels and bio-based products (Opgenorth et al., 2019). Growing interest in the DBTL cycle for metabolic engineering is largely due to improving capabilities in synthetic biology (e.g., synthetic biology tools, DNA synthesis, and genome editing), “omics” technologies, and Learning methods (Carbonell et al., 2018; Opgenorth et al., 2019; Robinson et al., 2020). The DBTL cycle uses synthetic biology to Design and Build genetic constructs in microbial hosts, after which the information gained from “omics” technologies, during the Test phase of the cycle, is passed on to Learning processes (Figure 3). What is Learned (e.g., COBRA models) is then fed back to new cycles of design to advance the engineering biology goal (Vavricka et al., 2020) for further strain development and optimization. Thus, facilitating the rapid optimization of microbial strains for production of any chemical compound of interest (Carbonell et al., 2018). Arguably the weakest link in the DBTL cycle workflow is the Learning process since mathematical models (of the engineered bioproduct, pathway, biological system, or biome) are only as good as their assumptions (Liu and Nielsen, 2019). Consequently, both high quality and large “omics” data sets are necessary to improve training models, ensuring increased accuracy and robustness of the Learning process.

Recently, Geiselman et al. utilized the DBTL cycle to engineer *Rhodosporidium toruloides*, an oleaginous yeast species with the ability to grow on lignocellulosic feedstock, to produce the diterpene ent-kaurene, a potential therapeutic, by the native mevalonate pathway and the non-native production of the diterpene precursor geranylgeranyl diphosphate (GGPP). Multi-omics data, in the first round of the DBTL cycle, suggested a limited availability of GGPP. In successive DBTL cycles, an optimal GGPP synthase (GGPPS) was selected, whose expression was balanced with the addition of kaurene synthase from *Gibberella fujikuroi* and a mutant version of farnesyl diphosphate (FPP) synthase from *Gallus gallus* that produces GGPP under strong promoters. The higher ent-kaurene titer achieved was the first demonstration of the production of a non-native diterpene from lignocellulosic hydrolysate in *Rhodosporidium toruloides* (Geiselman et al., 2020). Additionally, Opgenorth et al. used the DBTL cycle approach to optimize 1-dodecanol production in *E. coli* MG1655 strains by modulating ribosome binding sites and acyl-ACP/acyl-CoA reductase on a single operon. The proteomics and metabolomics data collected during the first DBTL cycle were used to train the Learning algorithms, with protein profiles being used to suggest the second DBTL cycle, which led to a 21% increase in 1-dodecanol titer. While this resulted in a 6-fold increase in what was previously reported, the study, however, highlighted the need for more accurate protein expression predictive tools and the importance of genomic sequencing checks on plasmids in cloning and production strains to establishing robust microbial cell factories (Opgenorth et al., 2019).

Adaptive laboratory evolution (ALE) studies the evolutionary forces and adaptive changes influencing microbial strain phenotypes, performance, and stability in order to acquire production strains containing beneficial mutations and positive traits (Dragosits and Mattanovich, 2013; Sandberg et al., 2019; Gibson et al., 2020). Microorganisms are cultured in a desired growth environment for an extended period of time, allowing natural selection to enrich for mutant strains with improved fitness (Johansen, 2018; Sandberg et al., 2019). Therefore, the throughput of ALE will depend on the experimental design. Furthermore, the task of identifying all beneficial mutations of an ALE experiment remains a major challenge for the field (Phaneuf et al., 2020). ALE is often used to optimize microbial growth rate, increase strain tolerance, improve stress regulation and adaptation, improve substrate utilization and uptake, increase product titer/rate/yield, as well as for biological discovery via systems biology, evolutionary modeling, and genome dynamics (Bergh, 2018; Dourou et al., 2018; Sun et al., 2018; Wang et al., 2018; Yan and Fong, 2018; Sandberg et al., 2019; Phaneuf et al., 2020). ALE experiments allow researchers to learn how to improve multiple strain properties simultaneously (Sandberg et al., 2019).

ALE has become a valuable tool in metabolic engineering for strain development and optimization by reliably facilitating microbial fitness improvements, via both predictable and non-intuitive mechanisms (Sandberg et al., 2019). ALE can be employed in the DBTL cycle in the Build step to recover strains with fitness issues or to optimize strains (Sandberg et al., 2019). Furthermore, ALE can be used in the Design step to enrich for mutant strains with improved fitness and can also replace both Design and Build steps in situations where a desired phenotype is tied to selection without the need for engineering (Sandberg et al., 2019; Lee and Kim, 2020). While ALE can precede the Test and Learn steps in the DBTL cycle, the information gained from these steps can also be utilized by ALE to produce strains with better properties. In this way, ALE may benefit from using “omics” technologies during the Test phase of the DBTL cycle (Horinouchi et al., 2018; Long and Antoniewicz, 2018; Walker et al., 2019; Wu et al., 2020).

In most microbial metabolic engineering studies, however, the Learn phase of the DBTL cycle is often addressed by hypothesis-driven user intuition that is often based on empirical evidence (Liu and Nielsen, 2019). As with the DBTL cycle, genomic sequence information in the traditional approach is invariably utilized in the initial stages of a study. However, genomics has come a long way in the last decade. Bar-seq can now be used to study population dynamics of *Saccharomyces cerevisiae* (*S. cerevisiae*) deletion libraries during bioreactor cultivation, enabling the identification of factors that impact the diversity of a mutant pool (Wehrs et al., 2020). Whereas, a sort-seq-guided engineering approach can be used to identify key mutated promotors for tuning expression levels, thereby facilitating the dynamic regulation of microbial growth as well as dynamic pathway regulation (Rohlhill et al., 2017). While transcriptomics yields gene expression data (i.e., activity of target genes, gene sequence data, and gene expression levels), proteomics and metabolomics approaches are increasingly being used for pathway analysis studies as they can measure protein translation and activity, respectively (Volke et al., 2019). Proteomics-guided approaches have been used to engineer polyketide biosynthesis platforms for aromatic compounds in yeast (Jakočiunas et al., 2020) and *in vitro* production of adipic acid (Hagen et al., 2016). In addition to this, metabolomics enables the assessment of pathway flux, carbon source diversion, and cofactor imbalance, which all contribute to the identification of pathway bottlenecks (Nielsen and Jewett, 2007; Zhao et al., 2020, Volke et al., 2019). Luo et al. used a metabolomics guided approach to characterize cannabinoid production in engineered *S. cerevisiae* and identified cannabinoid analogs produced by several promiscuous pathway genes (Luo et al., 2019). Furthermore, metabolomics analysis aided the design (Kang et al., 2016) and optimization (Kang et al., 2019) of a novel isopentenyl diphosphate-bypass mevalonate pathway in *E. coli* for C5 alcohol production. With a combined genetic, biochemical and fermentation approach, Uranukul et al. utilized the native glycolytic pathway in *S. cerevisiae* to produce monoethylene glycol, an important commodity chemical, and upon further metabolic engineering and process optimization were able to achieve 4.0 g/L (Uranukul et al., 2019). The integration of proteomics and metabolomics promises accurate assessment of pathway flux due to proper accounting of protein abundance. When pathway data is obtained in addition to transcriptomics data and/or large scale targeted/untargeted proteomics or metabolomics data, the impact of the engineered pathway on cellular metabolism and physiology can be determined. Exploring the interplay between heterologously expressed pathways and endogenous metabolism could reveal factors affecting strain variation, identify perturbed metabolic nodes, and produce new engineering targets (Chen, 2016).

### The Application of “Omics” Technologies to Agricultural and Food Biotechnology

Recent advances in agricultural biotechnology have led to new plant varieties being engineered by recombinant DNA technology and grown by farmers to respond to market demands and environmental challenges (https://www.usda.gov/topics/biotechnology). “Omics” technologies are being applied to agricultural biotechnology to enhance desirable phenotypic traits (e.g., color, taste, drought tolerance, pesticide resistance, etc.) (Aliferis and Chrysayi-tokousbalides, 2011; Van Emon, 2016). While “omics” plays a major role in improving crop quality, consistency, and productivity, they have also led to the development of food crops with enhanced nutritional composition (Van Emon, 2019) (Figure 2). Moreover, omics-driven systems biology provides an understanding of the interactions between the “omes” and mechanisms involved and provide links between genes and traits (Van Emon, 2016).

As arable land is being farmed more heavily, soil is becoming more susceptible to loss of structure, organic matter, minerals, and erosion. Thus, efforts are being made, via agricultural biotechnology, to maintain a sustainable supply of nutrients essential to the growth of crop plants. An integral part of this approach is the use of biofertilizers, which are the preparations containing specialized living organisms (i.e., microbial inoculants) that can fix, mobilize, solubilize, or decompose nutrient sources, and are applied through seed or soil to enhance nutrient uptake by plants (Mohanram and Kumar, 2019). The sustainable enhancement in food production from less available arable land relies on the balanced utilization of inorganic minerals, organic matter, and biofertilizer sources of plant nutrients to augment and maintain soil fertility and productivity. Widespread adoption of this approach, however, has been hindered by varying responses of microbial inoculants across fields and crops (Mohanram and Kumar, 2019). As a result, there is an urgent need to understand the mechanisms underlying the interdependencies between soil microbial communities and the host plant and their impact on crop productivity. These interactions are played out in the rhizosphere, which encompasses the region of soil that is directly influenced by root secretions and associated microbial communities (Zhalnina et al., 2018). In a recent comparative genomics and exo-metabolomics study, specific rhizosphere bacteria were shown to have a natural preference for certain aromatic organic acids exuded by plants, suggesting that plant exudation traits and microbial substrate uptake traits interact to yield the patterns of microbial community assembly (Zhalnina et al., 2018). Furthermore, the application of genomics and transcriptomics to the study of luxury phosphate uptake (i.e., the ability of microalgae to take up more phosphorus than necessary for immediate growth) revealed a range of Pi transporters in various microalgae and their expression patterns in relation to the availability of P (Yang et al., 2018; Mohanram and Kumar, 2019; Solovchenko et al., 2019). At present, “omics” technologies are being used to understand complex rhizospheric intercommunications, which is crucial to the development and choice of biofertilizer and, by extension, the construction of rhizopheres that promote stable plant growth, better crop productivity and yield (Mohanram and Kumar, 2019).

In the related field of food biotechnology, transcriptomics and metabolomics analysis showed that *Bacillus pumilus* LZP02 promote the growth of rice roots by enhancing carbohydrate metabolism and phenylpropanoid biosynthesis (Liu et al., 2020). Further, the application of “omics” to starch bioengineering is increasing our understanding of the specific contributions of the most important enzymes for starch biosynthesis. This has enhanced our ability to predict how starch-related phenotypes can be modified, thus ensuring further progress in the research field of rice starch biotechnology (Nakamura, 2018). “Omics” are solving issues surrounding food quality and traceability, to safeguard the origin of food, and discover biomarkers of potential food safety problems (Ferranti, 2018). In the wine industry, the wine microbiome associated with the fermentation of must has a great influence on factors transforming grapes to wine, including flavor and aroma. “Omics” characterization of the complex interactions between these microbes, the substrate and environment, is key to shaping wine production (Sirén et al., 2019). Finally, combining “omics” technologies with genome editing of food microbes can be used to generate enhanced probiotic strains, develop novel bio-therapeutics and alter microbial community structure in food matrices (Pan and Barrangou, 2020).

### The Use of “Omics” Technologies in the Development of Therapeutics for COVID-19

The coronavirus disease 2019 (COVID-19) characterized by the Severe acute respiratory syndrome coronavirus 2 [i.e., SARS-CoV-2, which binds to the ACE2 receptor in the lung and other organs (Ahmed et al., 2020)] has caused a global pandemic and slowed much of the world's economy. To date (December 5th, 2020), there are more than 64 million confirmed cases and 1.5 million confirmed deaths world-wide (https://www.who.int/emergencies/diseases/novel-coronavirus-2019). Thus, there is a pressing need for an effective countermeasure to mitigate the spread of the pandemic (van Doremalen et al., 2020).

Consequently, efforts are underway to fast-track the development and production of safe and effective vaccines against SARS-CoV-2. Prior knowledge of SARS and Middle East respiratory syndrome (MERS) has enabled scientists to target the spike protein as the viral antigen (via the ACE2 receptor). Moreover, the release of the SARS-CoV-2 genome sequence in January 2020 made it possible to expedite the development of next generation mRNA [e.g., *mRNA-1273 from NIH/Moderna* (Jackson et al., 2020) *and BNT162 from Pfizer/BioNTech* (Mulligan et al., 2020)] and DNA [e.g., ChAdOx1 nCoV-19 from University of Oxford/Vaccitech/AstraZeneca (Folegatti et al., 2020; van Doremalen et al., 2020)] vaccine platforms that encode for the antigen. Once injected into a host, the former (which is encased in lipid nano-particles) remains in the cytoplasm while the latter (which is encased in an attenuated adenovirus vector) enters its nucleus. The host cell translates these genetic materials into the spike protein, which decorates the surface of the cell and elicits an adaptive immune response mediated by T cells (e.g., CD4^+^ and CD8^+^) and B cells (i.e., antibodies). These vaccines were reported to be efficacious against SARS-CoV-2 in recent clinical trials, which underscores the importance of genomics to this new era of vaccine development.

Gordon et al. produced a SARS-CoV-2 protein interaction map via a proteomics-based approach to reveal targets for drug repurposing. They cloned, affinity tagged and expressed 26 of the 29 SARS-CoV-2 proteins in human cells and identified the associated proteins via proteomics analysis. A total of 66 human proteins or host factors were identified as possible drug targets of 69 compounds, from which two sets of these pharmacological agents showed antiviral activity. This work highlights the potential of host-factor-targeting agents, when acting alone or in combination with drugs that target viral enzymes, to be used as therapeutic treatments for COVID-19 (Gordon et al., 2020). Furthermore, computational immunoproteomics studies have the potential to guide lab-based investigations to evaluate specificity of diagnostic products, to forecast on potential adverse effects of vaccines and to reduce the use of animal models (Tilocca et al., 2020).

Recently, metabolomics was able to distinguish COVID-19 patients from healthy controls via the analysis of 10 plasma metabolites. Additionally, lipidomics data from this study suggests monosialodihexosyl ganglioside enriched exosomes could be involved in pathological processes related to COVID-19 pathogenesis (Song et al., 2020). Recent proteomics and metabolomics studies in COVID-19 patient sera suggest that SARS-CoV-2 infection causes metabolic dysregulation of macrophage and lipid metabolism, platelet degranulation, complement system pathways, and massive metabolic suppression (Shen et al., 2020); with the plasma metabolomic signatures appearing to be similar to those described for sepsis syndrome (Langley et al., 2013, 2014; Migaud et al., 2020). Furthermore, transcriptomics results indicate higher expression of genes related to oxidative phosphorylation both in peripheral mononuclear leukocytes and bronchoalveolar lavage fluid, suggesting a critical role for mitochondrial activity during SARS-CoV-2 infection (Gardinassi et al., 2020). Understanding the clinical presentation of COVID-19 as well as metabolomic, proteomic, and genetic profiles could lead to the discovery of diagnostic, prognostic and predictive biomarkers, ensuring the development of more effective medical therapy (Ahmed et al., 2020). Moreover, identifying metabolic biomarkers of severe vs. mild disease states in the lung during respiratory infections could lead to the development of novel therapeutics that modulate symptom and disease severity (Bernatchez and McCall, 2020; Shen et al., 2020). It is, therefore, critical to develop new approaches to early assess which cases will likely become clinically severe (Shen et al., 2020).

## Trends in “Omics” Related Biotechnology Research

**Figure 4:**
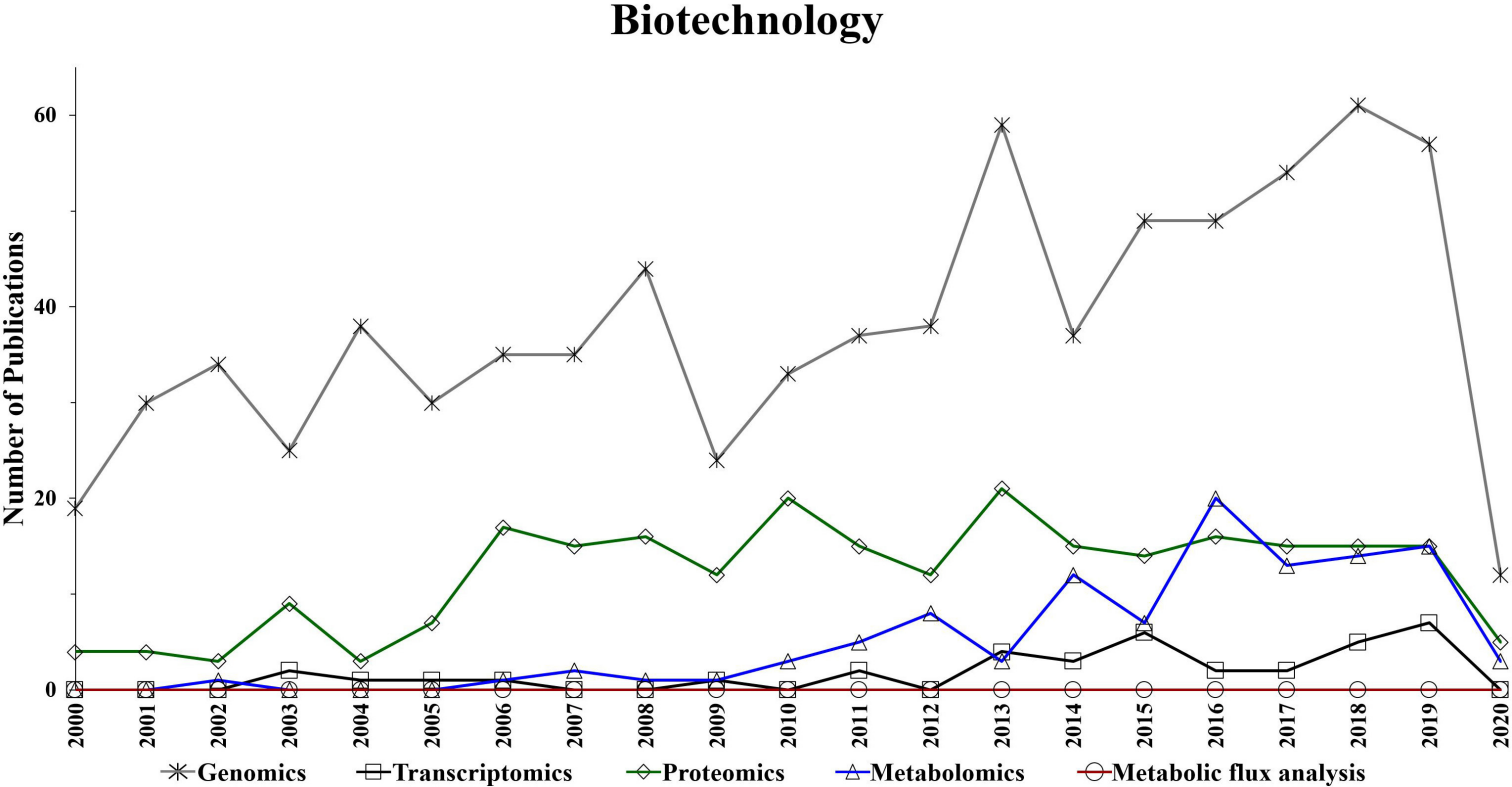
Number of annual publications that utilize “omics” technologies for biotechnology during the period of 2000–2020. Search criteria: the individual “omics” technology was selected. The search was conducted using the Web of Science platform.

**Figure 5:**
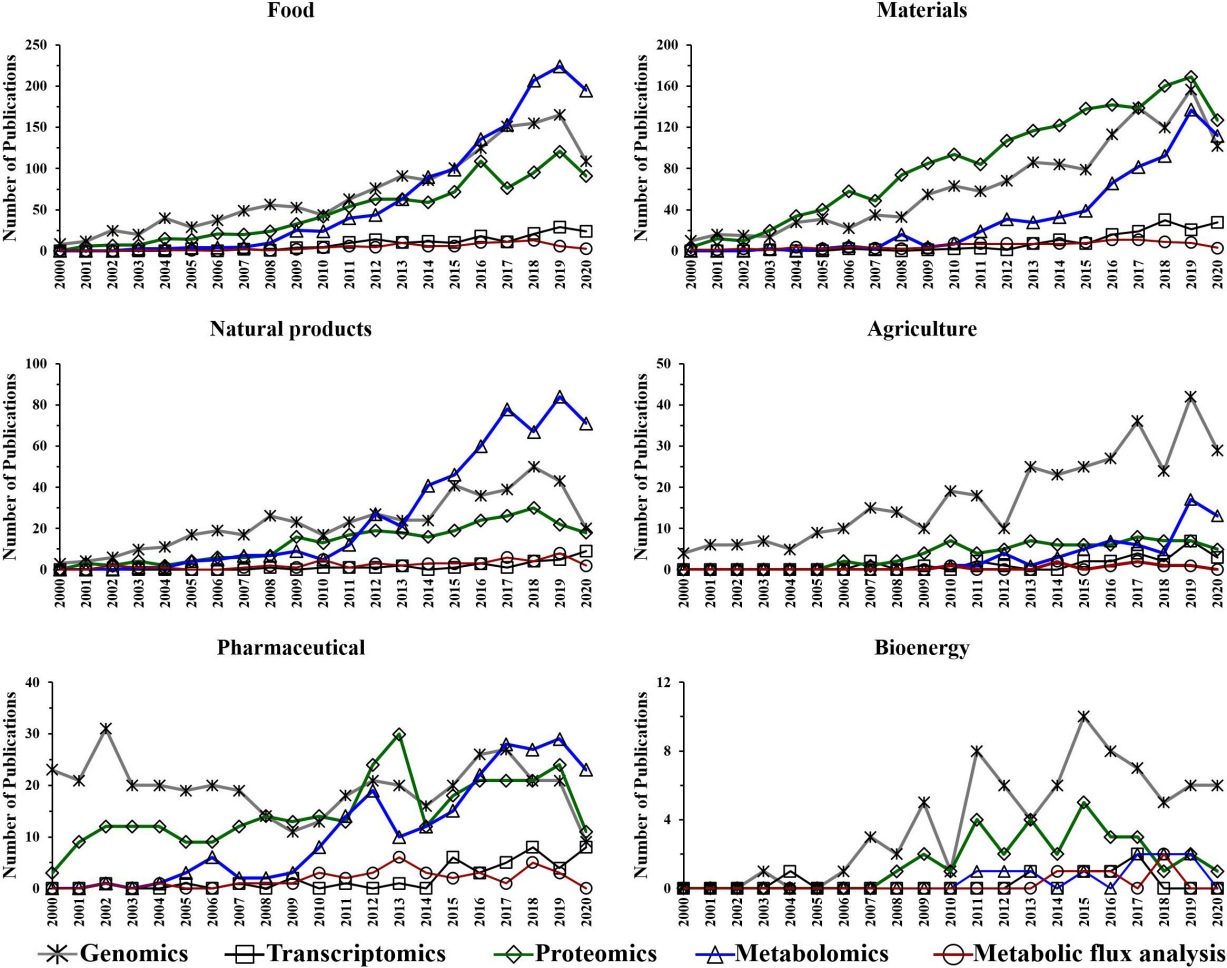
Number of annual publications that utilize “omics” technologies for biotechnology research areas during the period of 2000–2020. Search criteria: the individual “omics” technology and the individual biotechnology research area were selected. The search was conducted using the Web of Science platform.

The aim of this section is to present the trends in “omics” techniques utilization in biotechnology research (i.e., food, natural products, agriculture, pharmaceutical, materials, and bioenergy) during the course of last two decades. Such trends, which are based on the number of annual publications obtained from a search in Web of Science (www.webofknowledge.com) topics, are used to illustrate the progression of “omics” technologies in biotechnology. In Web of Science, the topics searched are as follows: title, abstract, author keywords, and keywords plus. It is worth noting that the trends in this section showing a reduction in the number of publications for the current year (i.e., 2020), which is expected to be a result of the COVID-19 related shut-down that has affected scientific labs worldwide and the mid-2020 collection of the data. Furthermore, it is also important to note that search entries, such as food, and bioenergy can generate publications based on the contributions of both closely and, to a lesser extent, loosely related topics. Additionally, it is worth noting that not all “omics” research data (e.g., industrial-based studies) is being published and made available to the public. Therefore, the trends data (Figures 4, 5) represents a qualitative rather than a quantitative measure of “omics” utilization.

Omics-based technologies serve as the connective tissue that links biotechnology to these fields of research. For example, the advancement in genomics technologies have improved biotechnology platforms, which have led to developments in pharmaceutical, bioenergy, food, materials, and agriculture research (Oksman-Caldentey and Saito, 2005; Crommelin et al., 2013; Misra et al., 2013; de Pablo et al., 2019; Rexroad et al., 2019). Figure 4 shows a steady growth in the number of genomics publications in biotechnology research, which might be due to the advancement in DNA sequencing, resulting in reduced cost and increased throughput (Pagani et al., 2012). Transcriptomics also appears to show a slight increase in the application of biotechnology-based research during the last decade (Figure 4).

Interestingly, while the utilization of proteomics in biotechnology seems to be significantly increased in the period of 2004–2006, it has generally plateaued during the following years (Figure 4). However, Figure 4 suggests a growing trend in the application of metabolomics studies to biotechnology-based research during the last decade. Additionally, metabolic flux analysis has also shown a slight growing trend in the last decade.

The performed search also suggests an increased utilization of genomics, proteomics and metabolomics during the last two decades in the following research fields: food, materials, and natural products (Figure 5). This is not surprising as there is a growing need for more phenotypic information. Consequently, scientists are using these “omics” techniques to facilitate their research. The improvements in proteomics and metabolomics analytical capabilities may also have contributed to the potential growth in their utilization over the last decade for those fields. Genomics has shown an upward trend in the number of publications for agriculture over the last 20 years (Figure 5).

The utilization of metabolic flux analysis in all research areas has shown a prospective slight growth trend during the last decade (Figure 5). The number of metabolic flux analysis publications, however, is relatively low in the perspective areas for similar reasons to that described for biotechnology.

## Conclusions

The DBTL cycle is becoming an increasingly adopted framework in metabolic engineering experiments as it provides a systematic and efficient approach to strain development. However, the DBTL cycle is limited by the Learning process since it requires high quality and large “omics” data sets to increase the accuracy and robustness of Learn methods. The DBTL cycle relies heavily on “omics” technologies during the testing phase of the cycle, and can be integrated into ALE experiments. “Omics” technologies have played major roles in the metabolic engineering of biofuels, bioproducts, and crop development. Proteomics and metabolomics are routinely applied to the analysis of engineered biosynthetic pathways in microbial hosts. Genomics sequencing information appears to be a key component in the development of next generation mRNA and DNA vaccines against virus's such as SARS-CoV-2. Whereas, transcriptomics, proteomics, and metabolomics analyses are being used to guide the development of therapeutic drugs for COVID-19. In the last 20 years, genomics has shown a steady growth in the number of biotechnology publications, however, the emergence of transcriptomics, proteomics, and metabolomics in this field of research is a testament to the development of robust “omics” technologies and methods.

## Author Contributions

BA conducted the data curation, data analysis, and literature review. EEKB conducted the data analysis and literature review. All authors contributed to the article and approved the submitted version.

## Conflict of Interest

The authors declare that the research was conducted in the absence of any commercial or financial relationships that could be construed as a potential conflict of interest.

## Footnotes

^1^*UniProt*. Available online at: https://www.uniprot.org/ (accessed September 30, 2020).
